# *Clostridium* and *Bacillus* Binary Enterotoxins: Bad for the Bowels, and Eukaryotic Being

**DOI:** 10.3390/toxins6092626

**Published:** 2014-09-05

**Authors:** Bradley G. Stiles, Kisha Pradhan, Jodie M. Fleming, Ramar Perumal Samy, Holger Barth, Michel R. Popoff

**Affiliations:** 1 Biology Department, Wilson College, 1015 Philadelphia Avenue, Chambersburg, PA 17201, USA; 2 Environmental Science Department, Wilson College, 1015 Philadelphia Avenue, Chambersburg, PA 17201, USA; E-Mail: kisha.pradhan@wilson.edu; 3 Department of Biology, North Carolina Central University, 1801 Fayetteville Street, Durham, NC 27707, USA; E-Mail: jodie.fleming@nccu.edu; 4 Venom and Toxin Research Programme, Department of Anatomy, Yong Loo Lin School of Medicine, National University Health System, National University of Singapore, Kent Ridge 117597, Singapore; E-Mail: dosrampe@nus.edu.sg; 5 Infectious Diseases Programme, Department of Microbiology, Yong Loo Lin School of Medicine, National University Health System, National University of Singapore, Kent Ridge 117597, Singapore; 6 Institute of Pharmacology and Toxicology, University of Ulm Medical Center, Albert-Einstein-Allee 11, Ulm D-89081, Germany; E-Mail: holger.barth@uni-ulm.de; 7 Bacteries Anaerobies et Toxines, Institut Pasteur, 28 Rue du Docteur Roux, Paris 75724, France; E-Mail: mpopoff@pasteur.fr

**Keywords:** *Clostridium*, *Bacillus*, protein, binary enterotoxin, receptor, actin

## Abstract

Some pathogenic spore-forming bacilli employ a binary protein mechanism for intoxicating the intestinal tracts of insects, animals, and humans. These Gram-positive bacteria and their toxins include *Clostridium botulinum* (C2 toxin), *Clostridium difficile* (*C. difficile* toxin or CDT), *Clostridium perfringens* (ι-toxin and binary enterotoxin, or BEC), *Clostridium spiroforme* (*C. spiroforme* toxin or CST), as well as *Bacillus cereus* (vegetative insecticidal protein or VIP). These gut-acting proteins form an AB complex composed of ADP-ribosyl transferase (A) and cell-binding (B) components that intoxicate cells via receptor-mediated endocytosis and endosomal trafficking. Once inside the cytosol, the A components inhibit normal cell functions by mono-ADP-ribosylation of globular actin, which induces cytoskeletal disarray and death. Important aspects of each bacterium and binary enterotoxin will be highlighted in this review, with particular focus upon the disease process involving the biochemistry and modes of action for each toxin.

## 1. Introduction

The *Clostridium* and *Bacillus* genera represent ubiquitous bacilli commonly found in soil, water, and gastrointestinal tracts of insects and animals, as well as humans. Both genera grow in low-oxygen environments; however, the clostridia are better adapted for anaerobic life with varying aerotolerance among different species. Pathogenic *Clostridium* and *Bacillus* species have developed unique mechanisms for survival within and outside of numerous host types, as evidenced by the various diseases frequently linked to their protein toxins and spores that include gas gangrene, food poisoning, antibiotic-associated diarrhea, pseudomembranous colitis, and enterotoxemia [1,2,3,4,5]. As subsequently described, a select group of bacterial binary enterotoxins can play pivotal roles in diverse diseases which also further accentuates the differences existing within this toxin family. The similarities, and dissimilarities, among these protein toxins suggest interesting evolutionary routes employed by some pathogenic *Clostridium* and *Bacillus* species. Common themes for these bacterial binary enterotoxins are: (1) the A and B components are secreted from the bacterium as separate proteins (not a holotoxin); and (2) enzymatic modification of globular (G) actin that destroys the filamentous (F) actin-based cytoskeleton and ultimately the intoxicated cell [6].

## 2. Pathogenic Bacilli and Binary Enterotoxins: Some of the Basics

The protein components of *C. botulinum* C2 toxin [7], *C. difficile* toxin (CDT) [8], *C. perfringens* ι-toxin and binary enterotoxin (BEC) [9,10], *C. spiroforme* toxin (CST) [11] as well as *B. cereus* vegetative insecticidal protein (VIP) [12] are produced as separate A and B molecules not associated in solution. Table 1 lists the gene locations and molecular weights of these toxin components.

**Table 1:** *Clostridium* and *Bacillus* binary enterotoxins and components.

Toxin Components	Gene Location	Protein M_r_ (kDa)
*C. perfringens* type E ι-toxin	140 kb plasmid [13]	
Ia		45 [13]
Ib		94 precursor [13]
		81 activated [13]
*C. perfringens* type A BEC	54.5 kb plasmid [10]	
BECa		47 [10]
BECb		80 [10]
*C. spiroforme* CST	chromosome [14]	
Sa		44 [11,14]
Sb		92 precursor [11,14]
		76 activated [11,14]
*C. difficile* CDT	chromosome [8]	
CDTa		48 [8]
CDTb		99 precursor [8]
		75 activated [8]
*C. botulinum* types C and D C2	chromosome [15] or 107 kb plasmid [16]	
C2I		49 [17]
C2II		81 precursor [15]
		60 activated [18]
*B. cereus* VIP	chromosome [19]	
VIP2		52 [20]
VIP1		100 precursor [20]
		80 activated [20]

The cell-binding components are enzymatically inert (as ascertained by existing assays) and produced as precursor molecules activated by various serine-type proteases like chymotrypsin or trypsin derived from the bacterium, host, or exogenous addition *in vitro* [21,22]. Loss of an *N*-terminal peptide (~20 kDa) from B precursor evidently causes conformational changes that facilitate oligomerization and subsequent docking with an A component(s).

The *Clostridium* and *Bacillus* binary enterotoxins are encoded by plasmid or chromosome-based genes with 27%–31% G + C content [23]. As just one specific example, the ι-toxin, there are two open reading frames with 243 non-coding nucleotides that separate the Ia and Ib genes. Mature Ia and Ib respectively consist of 400 and 664 amino acids [13]. The A and B components of *Clostridium* and *Bacillus* binary enterotoxins, except those for C2 or the recently described BEC, are respectively synthesized with a signal peptide of 29–49 and 39–47 residues [23]. The C2 and BEC toxins are uniquely linked to sporulation and released into the environment following sporangium lysis, thus obviating the need for a signal peptide and secretion [10,24]. It remains a curious mystery as to why similar, intestinal-acting toxins like the bacterial binary enterotoxins portrayed in this review are produced under quite different conditions (sporulation *versus* vegetative growth) by the same genus (*Clostridium*). Sequence identities between the sporulation-linked C2 and BEC components are only 29% and 41% for their A and B components, respectively [10].

Further comparisons of amino acid sequences among the *Clostridium* and *Bacillus* binary enterotoxin components reveal common evolutionary paths, as they share: (1) 80%–85% identity within the ι-toxin family; (2) 31%–40% identity between C2 and ι-family (ι, CDT, CST) toxins; and (3) 29%–31% identity between VIP and equivalent clostridial toxin components, which overall suggests that these toxin genes were derived from a common ancestor. Although unproven, it is plausible that the binary enterotoxin genes originated from an ancestral *Clostridium* and were horizontally transferred between *Bacillus* and *Clostridium* species via plasmids capable of inserting them into the bacterial chromosome, as evidenced by the CDT, CST, and C2 toxin genes. In fact, plasmid-borne genes for the ι and C2 toxins are flanked by insertion sequences [13,16,23]. In contrast, BEC appears unique and not simply a variant of these other binary toxins [10].

### 2.1. Clostridium perfringens: ι-Toxin and Binary Enterotoxin (BEC)

*C. perfringens* was first discovered in 1891 and consists of five serotypes (A–E), classically based upon four lethal, dermonecrotic toxins (α, β, ε and ι) neutralized by type-specific antiserum in animal assays [2,25,26,27]. Although not part of the typing scheme, sporulation-linked enterotoxin (*C. perfringens* enterotoxin or CPE) is also mouse lethal, causes erythema in guinea pigs, and linked to a major form of food poisoning found throughout the world [28,29]. Genetic methods involving multiplex PCR are now more commonly used than animal assays by many diagnostic laboratories for toxin typing of *C. perfringens* isolates [30,31,32,33].

The ι-toxin was initially described in 1943 by Bosworth [34], and its binary nature elucidated in the mid-1980s by exploiting cross-reaction and neutralization with *C. spiroforme* antiserum [9,35]. ι-toxin consists of iota a (Ia) and iota b (Ib). Individually, Ia or Ib are considered relatively nontoxic but together form a potent cytotoxin lethal to mice and dermonecrotic in guinea pigs [9,35,36]. Ia is an ADP-ribosyltransferase using nicotinamide adenine dinucleotide (NAD) to mono-ADP-ribosylate arginine [37], specifically R177, on muscle and non-muscle types of G-actin [6,38,39]. Ib, which lacks any discernible enzymatic activity, binds to a cell-surface protein(s) and subsequently translocates Ia into the cytosol of a targeted cell via acidified endosomes [40,41,42,43,44].

The ι-toxin is exclusively produced by type E strains and implicated in sporadic diarrheic outbreaks among calves as well as lambs [2,4,34,45,46,47]. Like the other binary enterotoxins described in this review, ι-toxin requires proteolytic activation as first described by Ross *et al*. in 1949 [21,47]. It was subsequently discovered, after cloning and sequencing of the ι-toxin gene, that proteolytic activation of Ib protomer (Ibp) into Ib occurs at A211 [13] which then facilitates Ia docking [42], formation of temperature- and voltage- dependent, cation-selective channels (K^+^ efflux, Na^+^ influx) [48,49], as well as SDS-stable heptamers on cell membranes [49,50] and in solution [40,49]. Further studies in artificial membranes and cells focused upon Ib-induced channels show: (1) 6-fold higher permeability for K^+^ *versus* Na^+^; (2) blockage by Ia only at pH ≤ 5.6; (3) decreased pH (to 4.6) does not open Ib channels, and in fact closes them (*i.e*., pH 3.7 shuts 50% of the channels); (4) that like the C2II component of C2 toxin, Ib-generated channels conduct various quaternary ammonium ions; and (5) that chloroquine does not block ι-induced channels, in contrast to those formed by C2II [48,51]. Ib heptamers generated in solution do not induce K^+^ release and are readily digested by pronase after binding to Vero (African Green Monkey kidney) cells at 37 °C, unlike Ib heptamers that form on the cell surface [49]. Like C2II, Vero cell-bound Ibp is not subsequently activated over time or after incubation with an excess of trypsin or chymotrypsin [50]. To date, extensive proteolytic activation studies similar to those for C2II and Ib have not been conducted with the other *Clostridium* and *Bacillus* binary enterotoxins.

For unknown reasons, pepsin, proteinase K, subtilisin or thermolysin activate Ibp more efficiently in solution than V8 protease, thrombin or even trypsin [21]. The zinc-dependent, lambda protease produced by some strains of *C. perfringens* also effectively activates ι-toxin, as well as the protoxin form of epsilon [52]. This makes sense as older cultures of *C. perfringens* type E generate proteolytically-activated ι-toxin [9,35,47]. Furthermore, the Ia molecule is also proteolytically activated by these same enzymes with a resultant loss of 9–13 amino acids (*N*-terminus), but it is still uncertain whether proteolysis of Ia increases: (1) docking efficiency to cell-bound Ib; (2) translocation efficiency into the cytosol; and/or (3) ADP-ribosyltransferase activity [21]. Proteolytic activation of Ia is seemingly unique among the *Clostridium* and *Bacillus* binary enterotoxins. It is noteworthy that amongst another family of AB toxins composed of heterologous proteins that form holotoxins in solution, such as *Escherichia coli* heat labile, *Shigella dysenteriae* shiga, and *Vibrio cholerae* cholera enterotoxins, the enzymatic A components are also processed by serine-type proteases. The difference with Ia is that these other A components form A_1_ and A_2_ subunits linked by a disulfide bond, subsequently reduced within the target cell’s endoplasmic reticulum [53,54,55,56,57].

Intriguingly, a study by Nagahama *et al*. suggests that dose-dependent binding of only Ib (≥100 ng/mL) to A431 (human epithelial) and A549 (human lung) cells (eight different lines tested) can cause rapid loss of ATP and cytotoxicity [58]. This is an interesting twist from the *Clostridium* and *Bacillus* binary enterotoxin paradigm not involving lipid-raft based oligomerization, unlike that previously described for ι-toxin by different groups using different cell lines [59,60]. It is possible that similar analysis with other binary enterotoxins portrayed in this review might yield equivalent results, thus promoting new ways of thinking.

In addition to the ι-toxin, Yonogi *et al*. recently describe a novel, binary enterotoxin of *C. perfringens* (BEC) produced by different type A isolates implicated in two food-borne gastroenteritis outbreaks in Japan [10]. This report reveals two components for BEC [BECa (47 kDa) and BECb (80 kDa)] which share no sequence similarity with the single-chain CPE, yet like CPE, are produced during sporulation. Cultural conditions for BEC production involve Duncan-Strong medium used specifically for sporulation of *C. perfringens*, in which these genetically-distinct outbreak isolates were CPE negative. Crude culture supernatants from either BEC-producing isolate cause fluid accumulation in rabbit intestinal loops and suckling mice.

BEC is coded on a large plasmid containing 55 open reading frames (ORFs), in which 39 ORFs encode for proteins of unknown function. A limited screen of 36 other *C. perfringens* isolates (human intestine) reveals only one that harbors the genes for BEC, suggesting minimal prevalence throughout nature, to date. There is respectively 36%–43% and 28%–44% amino acid sequence identity of BECb and BECa with complimentary components of other *Clostridium* and *Bacillus* binary enterotoxins. The sequence data suggest BEC to be a novel toxin, and not a variant of those previously described in the literature. Although biological activity on Vero cells and in suckling mice is optimal when recombinantly-produced and purified BECa and BECb are combined, BECb alone (≥1 μg) can cause fluid accumulation in mice. Furthermore, enterotoxic activity of culture supernatant from a parent strain is knocked out by targeting the *becB* gene. BEC, like all other *Clostridium* and *Bacillus* binary enterotoxins presented in this review, modifies G-actin via ADP-ribosylation using NAD as substrate.

### 2.2. Clostridium spiroforme Toxin (CST)

Like *C. perfringens*, the distinctly-coiled *C. spiroforme* also causes diarrhea (spontaneous or antibiotic-induced) but only in rabbits [1,61,62,63,64,65,66,67,68]. Although *C. spiroforme* was first isolated from human feces, correlation with human disease has not been definitively proven [69]. Rabbits are most susceptible to *C. spiroforme*-induced diarrhea during stressful periods that include lactation, old age, weaning, and an altered diet [64]. The Sa and Sb components of CST are respectively analogous to Ia and Ib of the ι-toxin, as first determined by crossed-immunoelectrophoresis and neutralization studies with *C. perfringens* type E antiserum [1,11,35,70].

During the late 1970s it was thought that *C. perfringens* was causing colony outbreaks because type E antiserum neutralized the cytotoxic effects of cecal contents from diarrheic rabbits [62,71,72,73,74]. *C. perfringens* type E was however not isolated, and in 1983 a strong correlation was established between the presence of disease and *C. spiroforme* [1,62,64]. A clever selection process for clostridial spores was employed by Borriello and Carman involving heat (80 °C/10 min) or ethanol (50%/1 h at room temperature) treatment of cecal contents [1]. PCR-based detection methods for *C. spiroforme* (16S rRNA) as well as the toxin (Sa and Sb) genes have now been published [75]. A vaccine consisting of formalin-toxoided, *C. spiroforme* culture supernatant has been used experimentally but subsequent efforts are lacking in the literature [76]. The importance of vaccine development is further suggested, as antimicrobials used for treating rabbit colonies during a *C. spiroforme* outbreak have become less effective against this pathogen [77].

### 2.3. Clostridium difficile Toxin (CDT)

*C. difficile* was first described in 1935 and considered normal, healthy intestinal flora in infants [78]. Relative to *C. perfringens* and *C. botulinum* outlined in this review, the toxin-linked pathogenicity of *C. difficile* was determined relatively recent (~35 years ago, like that for *C. spiroforme*). In the United States, *C. difficile* infections cost the healthcare industry and patients billions of dollars every year [79]. The rise of *C. difficile* as a nosocomial and community-acquired pathogen can be attributed to multiple factors that include smoking, hospital design, highly dynamic bacterial genome, long-term care residency, advancing age (>65 years, involving decreased immunity and increased visits to healthcare facilities), as well as use of proton-pump inhibitors and antibiotics [79,80,81]. In fact, *C. difficile* is attributed to more nosocomial infections than the highly-heralded strains of methicillin-resistant *Staphylococcus aureus* [82]. Treatment options are few but include ironically antibiotics (*i.e.*, vancomycin, metronidazole, or more recently fidaxomicin) plus experimental methods such as fecal flora transplant as well as intravenous immunoglobulins and vaccines that target *C. difficile* toxins [83,84,85].

*C. difficile* produces various toxins (*i.e*., toxins A and B) in addition to CDT, and represents a major cause of enterocolitis (*C. difficile* associated diarrhea, or CDAD) often nosocomially acquired following use of antibiotics [5,8,79,82,83,86]. It is possible for *C. difficile* to be pathogenic with only CDT, but not toxins A and B, further suggesting the pathogenic potential of CDT in humans [87]. However, in a common hamster model for *C. difficile* colitis, a toxins A/B negative-CDT positive strain colonizes but does not cause disease [88]. A pseudomembrane consisting of white blood cells, fibrin, mucin, dead cells (bacterial and host), as well as viable bacteria is the hallmark of severe disease caused by *C. difficile*. Recurring bouts of *C. difficile* colitis are particularly problematic for some unfortunate patients. There is a correlation between the presence of CDT (CDTa and CDTb) genes and increased disease severity (*i.e*., mortality) elicited by different “epidemic” strains (*i.e*., PCR ribotypes 023, 027, and 078) of *C. difficile* found throughout the world [86,89,90].

Besides humans, animals involved in food production (cattle, chickens, pigs, rabbits, sheep), wildlife (elephant), and even pets (cats, dogs) can be colonized by *C. difficile* and its transmission likely goes either human to animal or in reverse [91,92,93]. Furthermore, commercially available meats and vegetables can harbor *C. difficile* and thus represent other environmental sources for human colonization [94,95,96]. It is not only food, but water contaminated by *C. difficile* spores is also a concern before and after treatment [97,98]. Clearly, exposure to *C. difficile* can naturally occur throughout our everyday existence. One important issue not resolved is relative infectious dose of *C. difficile* spores needed to elicit human disease which hinges upon aforementioned factors, and likely others yet unknown.

To further understand relative amounts of CDT produced by *C. difficile* in the gastrointestinal tract and during culture, a monoclonal antibody (Mab)-based ELISA has been developed for detecting CDTb [99]. The findings suggest that *in vivo* production of CDT is ~20-fold higher in human feces *versus* *in vitro* culture, thus highlighting obvious (and unknown) differences existing between the intestinal tract and a broth tube. Furthermore, like ι-toxin and CST, CDT also shares much structural similarity (*i.e*., 80% and 82% amino acid sequence identity of CDTa (48 kDa) and CDTb (75 kDa) with *C. perfringens* Ia and Ib, respectively) [8,13,22,23,100]. These three toxins represent the ι-family that does not include *C. perfringens* BEC, *C. botulinum* C2 toxin, or *B. cereus* VIP.

Additional structural commonalities of CDT with other ι-family toxins are highlighted by interchangeable protein components that result in biologically-active chimeras [11,22,100,101]. Interestingly, *C. difficile*, *C. perfringens* and *C. spiroforme* are all associated with gastrointestinal diseases in humans and/or animals [1,4,5,34,86,102], and the synthesis of common binary enterotoxins with interchangeable protein components likely reveals a shared evolutionary path for these ubiquitous pathogens in a common niche. Although not described in the literature, it is plausible that co-colonization by two binary enterotoxin-producing species could result in chimeric-toxin induced damage to the gastric epithelium *in vivo*.

Regarding CDT prevalence among hospital/patient isolates in the United Kingdom and United States, *C. difficile* strains analyzed in the early 2000s respectively revealed only 6% and 16% containing both CDTa and CDTb genes [5,86,103,104]. Barbut and colleagues found an 11% prevalence rate of CDT-producing strains in a French hospital from 2000–2004 [89]. In another study from the Netherlands (2005), a survey of 17 hospitals revealed a higher (30%) presence of both CDT genes among *C. difficile* isolates [105]. Furthermore, this same study revealed that 36% of patients with CDAD were community (not nosocomial) acquired cases. A very recent Romanian study of CDAD cases at one hospital in Bucharest (2011–2012) discovered that 69% of *C. difficile* isolates were CDT positive [106]. Finally, increasing prevalence of CDT-positive strains has been noted over time in Italy [107]. Overall, these and other studies suggest a disturbing trend revealing the importance of CDT in *C. difficile* pathogenesis and increasing prevalence around the world.

### 2.4. Clostridium botulinum C2 Toxin

*C. botulinum* (*Bacillus botulinus*) was first described in 1895 following a food poisoning incident in Belgium [108]. Like *C. perfringens*, the neurotoxin types (A-G) of *C. botulinum* are classically determined by mouse lethal assays with toxin-specific antisera [4,26,109]. In contrast to the classic botulinum neurotoxins, the C2 toxin produced by types C and D lacks neurotoxicity but induces vascular permeability, necrotic-hemorrhagic lesions, as well as a lethal fluid accumulation in lungs and intestinal tracts of various animals [7,110,111,112,113,114,115,116,117,118]. C2 toxin is synthesized by *C. botulinum* during sporulation [24] and incorporated into the spore coat [119], which is akin to CPE and BEC of *C. perfringens* [10,28,120]. Pioneering work on the cell binding and translocation component (C2II), as well as enzyme component (C2I), of C2 toxin was initiated in the late 1970s. This effort by Dr. Ohishi’s laboratory was the first describing protein synergy employed by any *Clostridium* or *Bacillus* binary enterotoxin.

Trypsin activation of the 81-kDa C2II precursor into C2IIa (60 kDa) occurs between K181 and A182 [18], generating stable C2IIa homoheptamers in solution [121]. The C2IIa complex mediates biological effects on cells, in conjunction with an ADP-ribosyltransferase (C2I), that involve the formation of ion-permeable channels in lipid membranes [122]. Electron microscopy of C2IIa oligomers on lipid bilayers reveals annular heptameric structures with inner and outer diameters of 20–40 and 110–130 angstroms, respectively [121]. Although C2II precursor binds to cells, it will not dock with C2I or facilitate cytotoxicity [123,124].

There are intriguing physical (molecular weights and epitopes), as well as functional (cytotoxicity), variations between C2I and C2II components produced by different *C. botulinum* strains [125,126] that perhaps is not surprising from an evolutionary perspective. Comparable structural and functional data are lacking for the other *Clostridium* and *Bacillus* binary enterotoxins. Finally, following earlier reports that C2I possesses ADP-ribosyltransferase activity specific for arginine [127], the intracellular substrate of C2 toxin was identified in 1986 as actin via modification of R177 [128,129,130]. These results represent a ground-breaking discovery of a new family of bacterial ADP-ribosylating proteins that target the actin cytoskeleton.

### 2.5. Bacillus cereus Vegetative Insecticidal Proteins (VIPs)

In contrast to the aforementioned clostridial binary enterotoxins, the binary-based *B. cereus* VIPs target corn pests (*i.e*., Northern and Western corn rootworms) but neither animals nor humans [3,12,131]. Additionally, lepidopterans are evidently not affected by the binary VIPs [12]. The *B. cereus* VIPs are composed of VIP1 (~86 kDa cell-binding component) and VIP2 (~54 kDa ADP-ribosyltransferase that targets actin) produced during the growth, not sporulation, phase [12,19]. VIP1 and VIP2 do not share structural similarity with VIP3 produced by *Bacillus thuringiensis*, a protein that does kill various lepidopteran species evidently through pore formation in the midgut epithelium [132]. In addition to its insect killing properties, *B. cereus* can cause human food poisoning via other protein toxins [3,133] and is considered a nonlethal intestinal symbiont of various soil-dwelling insects such as roaches, sow bugs, and termites [134].

Overall, the prevalence of binary enterotoxins in two different genera and diverse species suggests a shared evolutionary success. The striking similarities, and dissimilarities, in structure and function of these *Clostridium* and *Bacillus* proteins will now be addressed below.

## 3. Protein Structure and Function

**Figure 1:**
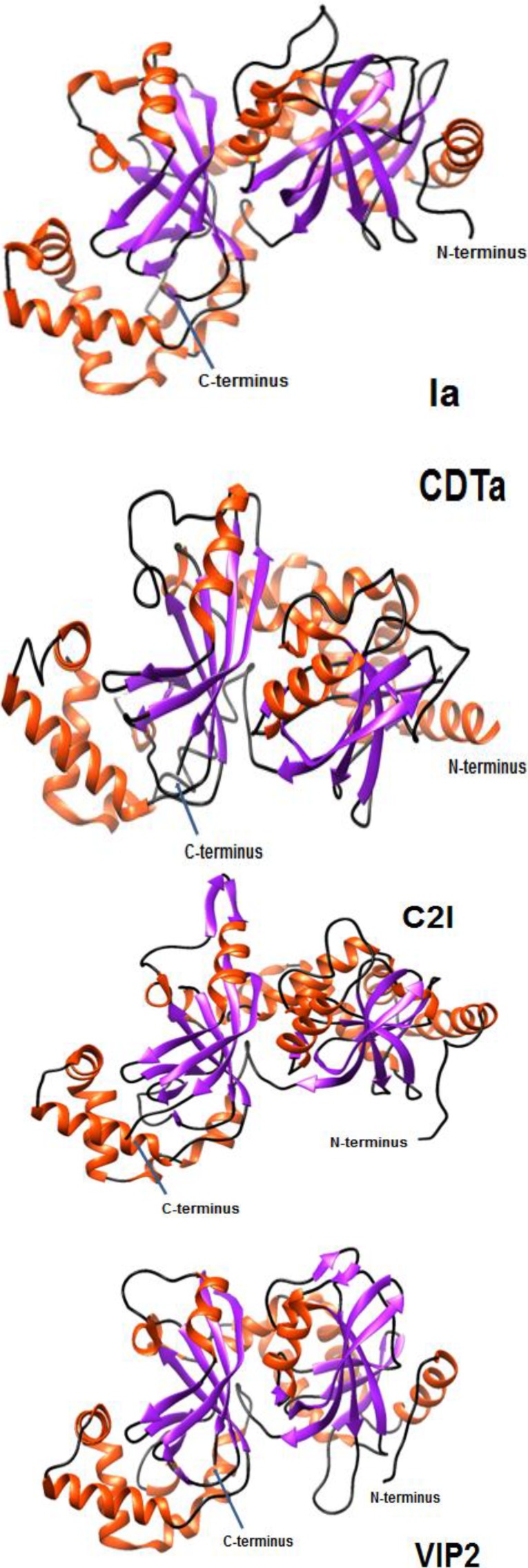
Ribbon plots of crystal structures for the A components from ι ([136]; Protein Data Bank (PDB) ID = 1GIQ), *C. difficile* toxin (CDT) ([137]; PDB ID = 2WN8), C2 ([135]; PDB ID = 2J3Z), and vegetative insecticidal protein (VIP) ([12]; PDB ID = 1QS1) toxins using Chimera (version 1.9) provided by the University of California, San Francisco, CA, USA [138]. Orange = alpha helix; Purple = beta sheet.

Crystal structures are available for the A components of ι, CDT, C2, and VIP toxins (Figure 1). All of these enzymes contain two domains, in which the *N*-terminus promotes interactions with the B component and the *C*-terminus possesses enzymatic activity. Use of PDBeFold (Protein Data Bank in Europe, version 2.59) reveals that the secondary structure of Ia, *versus* CDTa, C2I and VIP2, is respectively 93%, 69% and 82% matched along with Z scores of 19.8, 12.4 and 11.9. In contrast, for the B components there is only a low quality structure available for C2II [135]. Additionally, various studies have further investigated the structure and function of the C2, ι, and more recently CDT toxins by employing various techniques as follows.

### 3.1. C. botulinum C2II and C2I

Like the other *Clostridium* and *Bacillus* binary enterotoxins in this review, the *C*-terminus of C2II facilitates receptor-mediated binding on the cell surface, as deletion of only seven *C*-terminal residues effectively prevents C2IIa interactions with cells [18]. Antisera specific for the *C*-terminus (domain 4; residues 592–721), but not domains 1 (residues 1–264) or 3 (residues 490–592), block C2IIa binding to cells as determined by Western blots and cytotoxicity [18]. Neutralizing epitopes are sterically hindered after C2IIa-cell interactions, as addition of domain-4 specific antiserum does not afford neutralization. Deletion studies focused upon the *N*-terminus of C2II reveal that residues 1–181, lost after proteolytic activation of the C2II precursor, may be important for proper folding of the molecule [18]. Mutagenesis of the C2II gene within a conserved region of domain II (amino acids 303–331) results in a protein devoid of voltage-gating, but not chloroquine binding or translocation of C2I into the cytosol [139].

For the C2I molecule, residues 1–87 primarily mediate binding to C2IIa heptamers and translocation across the endosomal membrane [140]. Alignment of C2I amino acids 1–225 with VIP2 residues 60–275 reveals common, surface-exposed α-helices. In particular, residues 12–29 of C2I are akin to the first α-helical structure encompassing residues 71–85 in VIP2 [12]. Further analysis of component A crystals from different *Clostridium* and *Bacillus* binary enterotoxins shows two structurally similar domains possessing the same folding patterns, perhaps a result of gene duplication (Figure 1).

X-ray crystallography of both C2 toxin components has been reported at varying resolutions, evaluating particularly the conformational effects of different pH [135]. For C2I, there are minimal effects upon structure at pH 3 *versus* 6.1, which mimics endosomal acidification necessary for translocation of C2I through a C2IIa-created pore into the cytosol. As C2I has a minimum diameter of 40 angstroms, and the inner pore diameter formed by oligomeric C2IIa is maximally 32 angstroms, there is likely unfolding of C2I facilitated by low pH and contact with C2IIa. Orientations of the *N*- and *C*-terminal domains of C2I are the same as that discovered for Ia and VIP2 [12,135,136]. For C2II there is also little change in shape at pH 4.3 and 6.0; however, the *C*-terminal domain used for binding cell-surface receptor was not readily resolved and suggests plasticity [135].

Crystallography of other bacterial ADP-ribosyltransferases like *Bordetella pertussis* pertussis toxin [141], *Corynebacterium diphtheriae* diphtheria toxin [142], *E. coli* heat-labile enterotoxin [143], *Pseudomonas aeruginosa* exotoxin A [144], as well as VIP2 [12] reveals within the *C*-terminus: (1) two antiparallel β-sheets flanked by a pair of α-helices; and (2) a highly conserved catalytic domain containing an EXE motif found in prokaryotic as well as eukaryotic ADP-ribosyltransferases [6,13,23,136,145]. In contrast, overall sequence similarity of these toxins within the *C*-terminus is low. Studies focused upon the EXE motif of *C. botulinum* C2I show that an E387Q mutation prevents ADP-ribosyltransferase, but not NAD-glycohydrolase, activity while the same alteration of E389 inhibits both [146].

### 3.2. C. perfringens Ib and Ia

Like C2II, four distinct domains on Ib have also been described via deletion mutagenesis and antibody studies [50,147]. For instance, cleaving just ten amino acids from the *C*-terminus (domain 4) prevents Ib binding to Vero cells, and Ib peptides containing ≥200 *C*-terminal residues are competitive inhibitors of the toxin [147]. On the other end of Ib, deletion of just 27 *N*-terminal Ib residues from domain 1 prevents Ia docking without affecting cell binding of this construct that also effectively competes with ι-toxin *in vitro* [147]. In this same study, three Mabs targeting an *N*-terminal epitope (residues 28–66) had no effect upon Ib binding or cytotoxicity. These immunoreagents might not occupy the Ib site necessary for Ia docking, or perhaps oligomerization of Ib and/or docking of Ia readily displace these antibodies. In contrast, two Mabs recognizing unique Ib epitopes within *C*-terminal residues 632–655 afford protection against ι cytotoxicity. One Mab prevents Ib binding to cells while the other does not, yet the latter efficiently prevents Ib oligomerization on the cell surface [50,147]. Results for the latter Mab further reveal the importance of Ib oligomerization on biological activity of ι-toxin, as do studies with Ibp, a molecule that remains as a cell-bound monomer [42,50]. None of these Mabs recognize Ib on the cell surface, suggesting that epitopes are not accessible after binding to receptor. Furthermore, oligomerization of Ib also does not occur on Vero cells at 4 °C, although there is binding to the cell surface, while ι-toxin resistant cells (MRC-5, human lung) bind Ib without subsequent oligomerization [42,50]. The cumulative data clearly support Ib oligomerization for biological activity of ι-toxin.

All aforementioned Mabs against Ib recognize Ibp or *C. spiroforme* Sb in an ELISA and Western blot [147]. C2II is also recognized in an ELISA by one Mab that prevents Ib binding to cells, but C2 cytotoxicity is not neutralized *in vitro*. C2II and Ib bind unique receptors via their *C*-terminus and share little sequence similarity within this region [13,15,18,42,43,44,147,148,149], thus these distinct biological characteristics make this finding of Mab cross-reactivity quite curious.

There is also a calcium binding motif (DXDXDXXXDXXE) found within the *N*-terminus of B components from these binary enterotoxins, as well as in the distantly-related protective antigen of *Bacillus anthracis* edema and lethal toxins [150,151]. The proposed role played by chelation of calcium involves maintenance of protein conformation that affects A docking, as evidenced by ι-toxin [151].

To date, there is no crystal structure for Ib but that for Ia is available [136,150,152] (Figure 1). Analysis of Ia reveals two domains that share conformational, but little sequence, similarity. Other A components in this binary-enterotoxin family have the same structure, typical of ADP-ribosyl transferases targeting actin [152]. Further highlighting structural commonalities among these proteins is the catalytic C domain of Ia (residues 211–413) [13] and VIP2 (266–461) [12] that are quite similar, possessing 40% sequence identity and equivalent surface charges. One obvious difference between Ia and VIP2 is the spatial orientation of the first glutamic acid found within the conserved catalytic motif, 378EXE380 of Ia. Like C2I [146], the first glutamic acid within the EXE motif of Ia facilitates ADP-ribosyltransferase, but not NAD-glycohydrolase, activity [153]. Further analysis of Ia reveals that R295 and E380, which are conserved residues among ADP-ribosyltransferases [154,155,156,157], are also important for Ia catalysis [145,153]. The 338STS340 motif is located near the active site of Ia and other ADP-ribosyltransferases. Alanine replacement of any residue in the STS motif of Ia, especially the first serine, decreases enzymatic activity [153]. Extensive mutagenesis studies of Ia that focus upon the NAD binding cavity reveal that Y246 and N255 are important for ADP-ribosyltransferase, but not NAD-glycohydrolase, activity unlike Y251 involvement in both [153]. The binding of actin and NAD by Ia is accomplished by five loop structures, via ionic and van der Waals interactions [152]. All ADP-ribosyltransferases within the *Clostridium* and *Bacillus* binary-enterotoxin family (BECa, C2I, CDTa, Ia, Sa, and VIP2) target G-actin, which is a commonly conserved protein found throughout nature that plays a pivotal role in the cytoskeleton and homeostasis [6,8,10,38,39,70,100,128,152,158].

### 3.3. C. difficile CDTa and CDTb

In contrast to the C2 and ι toxins, less biochemical work has been done with the CDT components. Crystal data at different pH (4.0, 8.5 and 9.0) reveal conformational shifts for CDTa, particularly within the active site at low pH [137]. Structure-function studies show that the same amino acids are also necessary for enzymatic activity, in which Ia shares respectively 40% and 84% overall sequence identity with C2I and CDTa [13,23,101,146]. Amino acids necessary for ADP-ribosylation are similar between Ia (R295, R296, R352, Q300, N335, E378, E380) and CDTa (R302, R303, R359, Q307, N342, E385, E387) [137,145]. NAD and NADPH, sources of ADP-ribose for enzymatic transfer to G-actin, also uniquely make direct contact with S345 in CDTa which suggests differences in substrate interactions *versus* Ia [137]. The EXE motif of ADP-ribosyltransferases, part of the ADP-ribosyl turn-turn (ARTT) loop important for stabilizing substrate-enzyme complexes, differ regarding substrate contact made by Ia and CDTa [137,152,153]. The E385 and E387 of CDTa do not make contact with NAD or NADPH, unlike the equivalent E378 and E380 of Ia [136,137]. The STS motif found in CDTa is important in ligand binding and possibly catalysis.

Unlike the *C*-terminal, enzyme-critical similarities of A components for the *Clostridium* and *Bacillus* binary enterotoxins, the *N*-terminal domains can differ. For instance, Ia (residues 1–210) and VIP2 (60–265) contain only 20% sequence identity, dissimilar surface charges, and different conformations as Ιa contains an additional α helix (residues 61–66) [12,13,19,136]. Relative to enzymatic components of the other binary enterotoxins, the Ib docking region on Ia is more centrally located within the *N*-terminal domain (residues 129–257) [136,159], *versus* C2I residues 1–87 that interact with C2II (137) or CDTa residues 1–240 docking to CDTb [101]. Overall, these data reflect evolutionary variation within the *Clostridium* and *Bacillus* binary enterotoxins.

## 4. Intoxication Process

### 4.1. Toxin Binding to Cell

To access the cytosol and G-actin, the *Clostridium* and *Bacillus* binary-enterotoxin family must initiate intoxication via B components binding to a targeted cell via a receptor(s) to form a homooligomer complex. This acts as a platform for docking of A to the cell surface. To further understand the binding and oligomerization properties of B components on cell surfaces, studies have delved into the role(s) played by lipid rafts [59,60,160]. Lipid rafts are cholesterol-rich, detergent-insoluble (at 4 °C) “structures” or “microdomains” located on the cell membrane. These microdomains inadvertently serve as attachment, entry, and sometimes exit sites cleverly pirated by various bacteria, viruses, and toxins [161,162,163,164]. Results suggest that *C. perfringens* Ib, but not Ibp, localizes into these membrane microdomains on Vero cells that are susceptible to ι-toxin [59,60]. Comparable studies with C2IIa have been reported, and in fact the phosphatidylinositol 3-kinase pathway is necessary for C2 toxin internalization via lipid rafts [165]. Finally, the cell-surface receptor for CDT, CST and ι-toxin is lipolysis-stimulated lipoprotein receptor (LSR) which forms clusters in lipid rafts following binding of CDTb, precursor CDTb, or just the receptor binding domain (amino acids 677–876) of CDTb [43,160]. It appears that for ι, CDT and C2 toxins, lipid rafts are necessary for oligomer formation although monomeric B components can evidently bind to receptor located outside of these microdomains. To date, comparable studies have not been reported for the other *Clostridium* and *Bacillus* binary enterotoxins.

By using polarized CaCo-2 (human colon) cells, Blöcker *et al*. [40] discovered that the Ib receptor is primarily localized onto the basolateral membrane. Richard *et al*. [41] revealed that Ib traverses CaCo-2 cells from either the apical or basolateral surface and internalizes Ia found on the distal side, even when Ia is added 3 h after Ib. In this latter study, addition of Ib-neutralizing antiserum or Mabs with Ia on the opposite surface *versus* Ib does not affect ι cytotoxicity. Two different groups reveal that Ib rapidly binds to Vero cells at 37 °C and forms a large complex (>200 kDa) in less than 1 min that remains evident for at least 2 h [49,50]. Over time, Ib oligomers decrease on/in cells and tailing is evident in Western blots, suggesting lysosomal degradation of the oligomer into smaller protein species that appear on the plasma membrane [49,166].

These collective data [49,50] are relevant to earlier work by Sakurai and Kobayashi [36] showing that Ia injected intravenously into mice 2 h after Ib causes death, suggesting extended availability of Ib *in vitro* and *in vivo*. Furthermore, if toxin neutralizing antiserum targeting Ib is given just 5 min after an Ib + Ia injection, mice are not protected against lethality. In this same study it was also discovered that Ib given intradermally to guinea pigs, followed by intraperitoneal Ia, elicits a dermonecrotic lesion at the Ib injection site. Evidently, Ia remains in circulation for an extended period and is neutralized by antibody given 2 h after an Ia injection. In summary, Ia “locates” Ib in the body via general circulation, perhaps a common characteristic of not just ι but other *Clostridium* and *Bacillus* binary enterotoxins that could be exploited for therapeutic purposes? Comparable discoveries were reported by Simpson [118] for C2 toxin in mice and rats.

In regards to receptor binding, much work has been done in the past with C2II. The C2II precursor and proteolytically-activated C2IIa bind to intestinal cells and brush border membranes [167]; however, only C2IIa has hemagglutinating properties with human and animal erythrocytes, which is a process competitively inhibited by various sugars like *N*-acetylgalactosamine, *N*-acetylglucosamine, l-fucose, galactose, or mannose [168]. Trypsin or pronase pretreatment of human erythrocytes prevents C2IIa-induced hemagglutination, thus suggesting a glycoprotein receptor. Furthermore, chemical mutagenesis of CHO cells generated those resistant to C2 toxin because they lack *N*-acetylglucosaminyltransferase I activity necessary for synthesizing asparagine-linked carbohydrates [148,169]. These mutant cells are still susceptible to ι-toxin, providing evidence that C2IIa and Ib recognize unique receptors. Additionally, pretreatment of cells with various lectins or glycosidases does not affect Ib binding, suggesting that the receptor (or part of) is not a carbohydrate [42]. C2IIa and Ib form voltage-dependent channels in lipid membranes [48,122,139,170,171,172], likely employing hydrophobic and hydrophilic amino acids (*i.e*., C2II residues 303–331) for insertion into the membrane [139].

In contrast to C2IIa, which binds and facilitates C2I-mediated cytotoxicity in all tested vertebrate cells [123,149,168,169,173,174], the cell-surface receptor for Ib is not as ubiquitous [42]. Recent studies by Papatheodorou *et al*. very nicely reveal LSR as the cell-surface receptor for the ι-family toxins (CDT, CST, and ι) [43,160,175]. LSR is a transmembrane lipoprotein found in various tissues and naturally facilitates lipoprotein clearance and tight junction formation, as well as plays a critical role in cell development [43]. Additional studies by Wigelsworth *et al*. provide evidence that CD44 also promotes intoxication of these same toxins [44]. Like LSR, the CD44 glycoprotein is also a single-pass transmembrane protein with multiple functions. CD44 acts as a receptor for multiple ligands, transduces signals, and is exploited by certain bacteria and viruses for cell entry. These independent discoveries of LSR and CD44 were made using different methodologies that respectively include single-gene knockouts of human, near-haploid leukemia cells (HAP1) and proteomic analysis of lipid rafts from Ib-treated *versus* untreated Vero cells [43,44,176]. How LSR and CD44 might interact to affect ι intoxication remains unresolved to date.

Moreover, it was recently shown in cancerous breast epithelial cells that reduced LSR levels significantly decrease ι-toxin sensitivity while overexpression of CD44 conveys toxin resistance [177]. Overexpression of CD44 in LSR-expressing cell lines correlates with decreased, toxin-stimulated formation of lysosomes and cytosolic levels of ι-toxin. These data suggest that CD44 drives ι-toxin resistance through inhibition of endocytosis in cancerous breast epithelial cells and highlights the importance of cell-type specificity during intoxication. Discovery of LSR and CD44 involvement in ι-family intoxications provides invaluable insight towards potential therapeutics targeting: (1) the cell-surface interactions of these toxins; and (2) breast cancer cells. To our knowledge, receptor-binding studies for *B. cereus* VIP1 have not been reported in the literature.

### 4.2. A Docking to B and Internalization

*N*-terminal domains within the A and B components of each *Clostridium* and *Bacillus* binary enterotoxin are intimately involved in forming an AB complex on the cell surface. After binding to a surface receptor, intracellular-acting bacterial toxins use two major pathways for gaining entry into the cytosol. There is retrograde routing through the Golgi apparatus and endoplasmic reticulum, employed by *S. dysenteriae* shiga [53,178] and *V. cholerae* cholera [56,179] toxins, which is inhibited by brefeldin A that subsequently causes protein accumulation within the endoplasmic reticulum [180]. The other route exploited by bacterial toxins involves translocation from acidified early endosomes into the cytosol, like that employed by diphtheria toxin [181], *B. anthracis* edema/lethal toxins [182], and the *Clostridium*/*Bacillus* binary enterotoxins described in this review. Subsequent transport of vesicles from early to late endosomes involves microtubules depolymerized by nocodazole, which inhibits trafficking into late endosomes [40,121,183,184]. Neither brefeldin A nor nocodazole influence the biological activity of C2 or ι toxins on cells. However, translocation of A components for C2, ι, or the edema and lethal toxins across the endosomal membrane is blocked by bafilomycin A, which inhibits vacuolar-type ATPases that acidify the endosome [40,121,185,186]. Decreased pH evidently induces conformational changes that promote membrane insertion of the heptameric B complex, followed by translocation of the A component(s) across the endosomal membrane. This is a process mimicked on the cell surface by simply lowering media pH [40,51,121,172,181,185,187]. It is not clear if B heptamers of these *Clostridium* and *Bacillus* binary enterotoxins enter the cytosol with the A components or remain attached to the endosomal membrane, possibly recycling to the cell surface in a degraded form [41,124,166].

**Figure 2:**
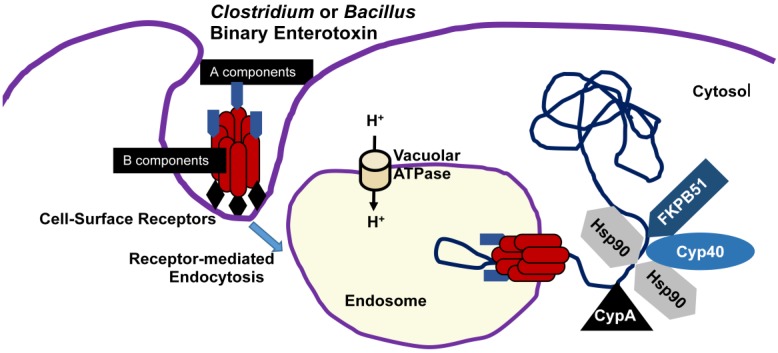
Basic model showing cell-surface binding and internalization of *Clostridium* and *Bacillus* binary enterotoxins.

To leave the endosome and enter the cytosol, A components of C2, CDT, and ι toxins traverse the endosomal membrane via chaperones such as heat shock protein 90 (Hsp90) and protein-folding enzymes that include the peptidyl-prolyl *cis/trans* isomerases cyclophilin A [188,189,190], cyclophilin 40 [191], and FK506 binding protein (FKBP) 51 [192]. Hsp90 is a highly conserved ATPase produced by all eukaryotic cells that provides an essential housekeeping role by regulating various proteins associated with cell signaling [193]. Inhibitors of Hsp90 (geldanamycin, radicicol), cyclophilins (cyclosporine A), and FKBPs (FK506) effectively delay C2-, CDT-, or ι-induced cytotoxicity because they block the pH-dependent translocation of A components into the cytosol (Figure 2). The inhibitors have no effects upon ADP-ribosyltransferase activity, binding to cell-surface receptor(s), or endocytosis.

Although unproven, the A components of *Clostridium* and *Bacillus* binary enterotoxins likely unfold and thread through toxin-generated channels in the membrane into the cytosol as proposed for diphtheria toxin [194]. Ratts *et al*. [195] report that Hsp90 and thioredoxin reductase, found in a cytosolic complex, are both required to transport diphtheria toxin from the endosome. Intriguingly different is that geldanamycin and radicicol are necessary for inhibiting diphtheria cytotoxicity, whereas either drug alone inhibits CDT, C2, or ι cytotoxicity. Thioredoxin reductase might cleave the disulfide bond between the A and B chains of diphtheria toxin, like that for the *C. tetani* tetanus toxin and *C. botulinum* neurotoxin A [196]. However, disulfide bonds and reductive activation have never been described for any of the *Clostridium* and *Bacillus* binary enterotoxins.

### 4.3. ADP-Ribosylation: Destruction of the Actin Cytoskeleton, Intoxicated Cell, and Perhaps the Host

Once inside the cytosol, the A component can mono-ADP-ribosylate G-actin that subsequently disrupts F-actin formation and the cytoskeleton. The basic mechanism of ADP-ribosylation employed by BEC, C2, CDT, CST, ι and VIP toxins is remarkably conserved by diverse bacteria from many different genera. All known ADP-ribosylating toxins use NAD as a source of ADP-ribose. There are at least four bacterial groups of ADP-ribosylating toxins based upon intracellular targets, that include: (1) elongation factor 2 modified via histidine variant (diphthamide) by diphtheria toxin and exotoxin A through an *N*- and *C*-terminal active site, respectively; (2) heterotrimeric G-proteins modified via cysteine by pertussis toxin, or arginine by *E. coli* heat labile enterotoxin and cholera toxin through an *N*-terminal active site; (3) Rho and Ras GTPases respectively modified via asparagine by *C. botulinum* C3 exoenzyme and arginine by *P. aeruginosa* exoenzyme S through a *C*-terminal active site; and (4) G-actin modified via arginine through a *C*-terminal active site. This last group includes *B. cereus* VIP [12,19,131], *C. botulinum* C2 toxin [128,129,130,197], and the ι-toxin family of *C. difficile* CDT [100,101], *C. perfringens* ι-toxin [38,39,136,152,156,198] as well as *C. spiroforme* CST [70,199]. From a sequence similarity perspective, *C. perfringens* BEC appears to be distinct from all other *Clostridium*/*Bacillus* binary enterotoxins and the actin residue modified has not been identified to date [10].

Actin (~42 kDa, G monomer) is found in all eukaryotic cells and structurally conserved between diverse species [200,201]. Many important eukaryotic efforts depend upon actin and include maintenance of cell structure, homeostasis, as well as the immune system. However, some pathogenic viruses and bacteria exploit the actin cytoskeleton for cell entry, intra- and inter-cellular movement, and in the case of *Clostridium* and *Bacillus* binary enterotoxins, targeting of the actin cytoskeleton causing cell death [6,201].

The actin-ADP-ribosylating enterotoxins of *Clostridium* and *Bacillus* species can be subdivided into two groups. C2 toxin, the first bacterial toxin discovered to mono-ADP-ribosylate actin, exclusively modifies R177 on β/γ-nonmuscle, as well as γ-smooth muscle, isoforms [6,128,129,130,202,203,204,205,206]. The ι-family toxins are less discriminating and mono-ADP-ribosylate R177 found on all G-actin isoforms, that includes skeletal muscle [39,206]. Perhaps varying substrate specificities lie in CDTa, Ia, and Sa having an actin-binding sequence of LKDKE *versus* LKTKE for C2I [6,8,13,23,152,207]. Modification of actin by *C. perfringens* BEC likely occurs at R177, but this remains unconfirmed along with recognition of different actin isoforms [10]. F-actin does not represent a substrate target for any of these bacterial binary toxins, but ADP-ribosylation of G-actin inhibits assembly into F-actin strands and cytoskeletal development [6,129,136,197,202,203,204,208,209]. From a bacterium’s perspective for survival, disruption of a eukaryote’s cytoskeleton can prevent phagocytosis [210] and intracellular trafficking of vesicles necessary for homeostasis. Furthermore, the ι, CDT and C2 toxins induce microtubule-based protrusions from epithelial cells in just 90 min after toxin exposure [211,212]. Length of these cellular extensions is concentration dependent, as lower amounts of toxin cause longer protrusions. These actin-free protrusions respectively promote *C. difficile* adherence ~5- and 4-fold to intoxicated Caco-2 cells and intestinal tract of gnotobiotic mice, probably slowing bacterial elimination from the intestinal lumen as well as increasing toxin-based damage to the mucosa [211,212]. In particular, CDT reroutes vesicles containing fibronectin from the basolateral membrane to apical protrusions that ultimately promote bacterial adherence [212]. This was evident on cultured cells treated with CDT and in murine intestines (cecum and colon epithelium) following infection by CDT-producing *C. difficile* (ribotype 027). Correspondingly, intracellular levels of fibronectin are decreased in Caco-2 cells following CDT or C2 toxin exposure. The authors reveal that secretion of fibronectin from an intoxicated cell is dependent upon matrix metalloproteases and calcium signaling [212].

Ultimately, a susceptible cell exposed to a sufficient dose of *Clostridium* or *Bacillus* binary enterotoxin dies and releases valuable nutrients into the environment, that become readily available for bacterial consumption. From a research perspective, toxins that modify actin are invaluable tools for studying the cytoskeleton and numerous cell processes during both homeostasis and disease progression.

## 5. Conclusions

As presented in this review, the enteric-targeting binary toxins from various *Clostridium* and *Bacillus* species possess unique characteristics yet commonly target the cytoskeleton, specifically actin. Historically, the discovery of *C. perfringens* ι-toxin in 1943 was the first for any *Clostridium* or *Bacillus* binary enterotoxin [34]. Subsequently, the multi-component nature of *C. botulinum* C2 toxin, *C. perfringens* ι-toxin, *C. spiroforme* CST, *C. difficile* CDT, *B. cereus* VIP, and *C. perfringens* BEC were respectively elucidated in 1980 [7], 1986 [9,35], 1988 [70], 1997 [8], 1999 [12], and 2014 [10]. There have been many subsequent studies exploring the biochemistry and biological effects for some of these toxins. Clearly there is more work to be done, particularly involving the evolution and therapeutic potential of these enterotoxins.

Further exploration by researchers employing gene probes and specific toxin antibodies will likely unveil new binary toxins produced by other bacteria, and perhaps those from different genera. As one example of very recent discovery, *C. perfringens* BEC was isolated in Japan from human food-poisoning strains of type A [10]. This toxin, although employing a basic binary mechanism, is also structurally distinct from all of the other *Clostridium* and *Bacillus* binary enterotoxins described in this review. Further study will perhaps unveil intriguing insight involving the evolutionary paths taken by binary enterotoxin-producing *Clostridium* and *Bacillus* species. Finally, it is evident that a knowledge-based understanding of the past will foster additional creative efforts, by various international groups, targeting these fascinating proteins.
